# Unlocking the future of hepatocellular carcinoma treatment: A comprehensive analysis of disulfidptosis-related lncRNAs for prognosis and drug screening

**DOI:** 10.1515/med-2024-0919

**Published:** 2024-04-05

**Authors:** Haojun Wang, Wei Wang

**Affiliations:** Beijing Chaoyang Hospital, Capital Medical University, Beijing, 100020, China; Capital Medical University, Beijing, 100071, China

**Keywords:** hepatocellular carcinoma, disulfidptosis, lncRNA, prediction model, immune microenvironment

## Abstract

**Background:**

The disulfide stress-induced cell death known as disulfidptosis is characterized by the disintegration of cytoskeletal proteins and F-actin as a result of an excessive buildup of disulfides within the cell. The relationship between disulfidptosis-associated long non-coding RNA (lncRNA) in hepatocellular carcinoma (HCC) progression is still not clearly understood. In this article, we aim to explore the crucial role of lncRNA in HCC.

**Methods:**

We initially obtained lncRNA related to HCC and clinical data from TCGA. The genes associated with disulfidptosis were identified through co-expression analysis, Cox regression, and Lasso regression. Additionally, we established a prognostic model for verification.

**Results:**

The risk model constructed with disulfidptosis-related lncRNA has been confirmed to be a good predictor of high and low-risk groups of HCC patients through survival curves, independent prognostic analysis, concordance index (C-index), ROC curves, and Kaplan–Meier plots. We also discovered differences in the response to immune targets and anticancer drugs between the two groups of patients, with GDC0810, Osimertinib, Paclitaxel, and YK-4-279 being more effective for patients in the high-risk group.

**Conclusion:**

In conclusion, we have developed a risk model that can guide future efforts to diagnose and treat HCC.

## 1 Introduction

Hepatocellular carcinoma (HCC) is one of the most prevalent and aggressive malignancies globally, posing a significant burden on public health systems. Despite advancements in diagnosis and treatment, the clinical outcomes for HCC patients remain diverse, necessitating the exploration of novel biomarkers for prognosis prediction and therapeutic intervention. In recent years, long non-coding RNAs (lncRNAs) have gained increasing recognition as pivotal regulators in a diverse array of biological processes, including cancer development and immune system function [1,2]. Among these, the emerging concept of “disulfidptosis” has garnered attention due to its intricate involvement in redox homeostasis and cellular fate determination [3,4]. Disulfidptosis, a recently identified process involving perturbations in cellular redox balance, has been shown to influence tumor initiation, progression, and therapeutic resistance in multiple cancers, including HCC [5,6]. In the tumor microenvironment (TME), cells undergo a complex and subtle adaptive process involving oxidative stress, dynamic changes in disulfide bonds, and the participation of redox-sensitive molecular mechanisms. Oxidative stress arises from the accumulation of an excessive amount of reactive oxygen species, possibly due to the rapid proliferation and heightened metabolism of cancer cells, as well as the low oxygen supply in the microenvironment [7,8]. This situation triggers dynamic alterations in intracellular disulfide bonds, which are not only associated with the structure and function of proteins but also regulated by the cellular redox status. Notably, long non-coding RNAs have emerged as integral regulators of disulfidptosis-associated pathways, thus exerting a substantial influence on HCC progression and patient outcomes [9,10]. This study aims to elucidate the role of disulfidptosis-related lncRNAs in predicting prognosis and immune activity in HCC. By integrating high-throughput transcriptomic data and clinical information from a well-defined cohort of HCC patients, we present a comprehensive disulfidptosis-related lncRNA signature. This signature not only holds promise as a robust prognostic tool for predicting patient survival but also provides insights into the intricate crosstalk between oxidative stress, tumor progression, and immune modulation.

In this article, we describe the methodology employed for the identification and validation of the disulfidptosis-related lncRNA signature. Furthermore, we present a detailed analysis of the association between this signature and key clinicopathological parameters, immune cell infiltration, and immune checkpoint expression within the HCC TME. Our discoveries illuminated the potential of disulfidptosis-related lncRNAs as biomarkers for patient stratification and guiding therapeutic decisions, thereby paving the way for personalized approaches in managing HCC. In our study, we conducted screenings encompassing drug resistance and anti-tumor compounds. Intriguingly, we identified significant correlations between drug sensitivity and the high-risk/low-risk groups across a variety of medications.

In conclusion, the integration of disulfidptosis-related lncRNA signature holds significant potential in advancing our understanding of HCC pathogenesis, prognosis prediction, and immune modulation. New therapeutic approaches that target disulfidptosis-associated pathways to improve clinical outcomes in HCC patients may be possible because of the knowledge gathered from this work.

## 2 Materials and methods

### 2.1 Data acquisition

We obtained RNA sequencing data, detailed clinical information of patients, and somatic mutation data for HCC from the TCGA database. During the data selection phase, we exclusively considered samples that met specific criteria, excluding any potential confounding factors that could influence the results. In the preprocessing steps, we performed normalization and denoising procedures to ensure the consistency and quality of the data. All HCC patients were meticulously divided into a training group (*n* = 185) and a test group (*n* = 185), and this division was carried out randomly. The collection and processing of all data were strictly in accordance with the guidelines stipulated by TCGA. Moreover, we referenced the genes related to disulfidptosis from the most recently published literature [11].

### 2.2 A risk model related to disulfidptosis

Pearson correlation analysis served as the initial approach to identify DRlncRNAs by evaluating the correlation between lncRNAs and the disulfidptosis process. The threshold for correlation strength was set at |*R*| 0.5, ensuring the selection of lncRNAs with a substantial correlation, and the significance level was rigorously set at *p* < 0.001, emphasizing the robustness of the correlations selected. Following the correlation analysis, dimension reduction techniques were applied to pinpoint the most influential DRlncRNAs for prognosis. Multivariate Cox analysis, a statistical method accounting for multiple variables simultaneously, identified four key DRlncRNAs associated with HCC prognosis. To further refine the model, univariate Cox analysis and the Lasso algorithm were employed, leveraging their abilities to enhance dimensionality reduction and identify the most relevant predictors for the prognostic model. The integration of these refined DRlncRNAs into a predictive model allowed for the assignment of a risk score to each HCC patient, providing a quantitative measure of their prognostic risk. Based on the median risk score, patients were subsequently categorized into two distinct groups, allowing for a stratified analysis of prognosis. This risk stratification approach enhances the precision of prognostic assessments, enabling a more nuanced understanding of patient outcomes in the context of the identified DRlncRNAs.

### 2.3 Risk characteristics of HCC prognosis

Utilizing chi-square tests, ROC curve analysis, and Kaplan–Meier survival analysis, we investigated prognostic characteristics. Nomograms, clinical risk variables, and survival time predictions were all made using dRlncRNA risk scores. In order to gain a deeper understanding of the ability of the nomogram model to make accurate predictions, we devised both the concordance index (C-index) and calibration curves.

### 2.4 Principal component analysis (PCA), gene ontology, and functional enrichment analysis

By conducting PCA, we can delineate the expression patterns of crlncRNA. Simultaneously, the relationships between three variables within the samples can be visually illustrated using a 3D scatter plot. Following that, a differential expression gene (DEG) analysis will be performed to further explore variations between different samples. Finally, through gene ontology (GO), kyoto encyclopedia of genes and genomes (KEGG), and gene set enrichment analysis (GSEA), we can delve into the relevant biological pathways and functions.

### 2.5 TME, somatic mutation analysis, and drug prediction

TME variation is quantified by the ESTIMATE method. The CIBERSORT method determines how these groupings compare across 22 immune cell types. The ssGESA algorithm classifies individuals into risk categories based on their immune system responses. To measure immune evasion by tumor cells and their response to immune checkpoint inhibitors, researchers developed the tumor immune dysfunction and exclusion score. Tumor mutational burden (TMB) is used to categorize patients with HCC into low- and high-risk categories based on TCGA data. Drug response can be predicted in HCC patients across risk groups using the “oncoPredict” R program, which generates IC_50_ values for commonly used anti-cancer medications.

### 2.6 Statistical analysis

All statistical methods and graphics were conducted using R (version 4.3.0), and statistical significance for all analyses was denoted with *p* < 0.05.

## 3 Results

### 3.1 Grouping and clinical information

Table 1 displays the clinical data for HCC for the 370 patients who were split into a training group (*n* = 185) and a testing group (*n* = 185). The results demonstrated that there were no differences in any clinical features between the training and testing groups.

**Table 1:** Characteristics of HCC patients

Covariates	Type	Total	Test	Train	*p* value
Age	≤65	232(62.7%)	115(62.16%)	117(63.24%)	0.9144
Age	>65	138(37.3%)	70(37.84%)	68(36.76%)	
Gender	FEMALE	121(32.7%)	61(32.97%)	60(32.43%)	1
Gender	MALE	249(67.3%)	124(67.03%)	125(67.57%)	
Grade	G1	55(14.86%)	26(14.05%)	29(15.68%)	0.8717
Grade	G2	177(47.84%)	88(47.57%)	89(48.11%)	
Grade	G3	121(32.7%)	63(34.05%)	58(31.35%)	
Grade	G4	12(3.24%)	5(2.7%)	7(3.78%)	
Grade	Unknown	5(1.35%)	3(1.62%)	2(1.08%)	
Stage	Stage I	171(46.22%)	91(49.19%)	80(43.24%)	0.4885
Stage	Stage II	85(22.97%)	42(22.7%)	43(23.24%)	
Stage	Stage III	85(22.97%)	42(22.7%)	43(23.24%)	
Stage	Stage IV	5(1.35%)	1(0.54%)	4(2.16%)	
Stage	Unknown	24(6.49%)	9(4.86%)	15(8.11%)	
T	T1	181(48.92%)	95(51.35%)	86(46.49%)	0.4902
T	T2	93(25.14%)	48(25.95%)	45(24.32%)	
T	T3	80(21.62%)	34(18.38%)	46(24.86%)	
T	T4	13(3.51%)	6(3.24%)	7(3.78%)	
T	Unknown	3(0.81%)	2(1.08%)	1(0.54%)	
M	M0	266(71.89%)	137(74.05%)	129(69.73%)	0.5832
M	M1	4(1.08%)	1(0.54%)	3(1.62%)	
M	Unknown	100(27.03%)	47(25.41%)	53(28.65%)	
N	N0	252(68.11%)	133(71.89%)	119(64.32%)	1
N	N1	4(1.08%)	2(1.08%)	2(1.08%)	
N	Unknown	114(30.81%)	50(27.03%)	64(34.59%)	

### 3.2 Construction of dRlncRNAs risk model

**Figure 1:**
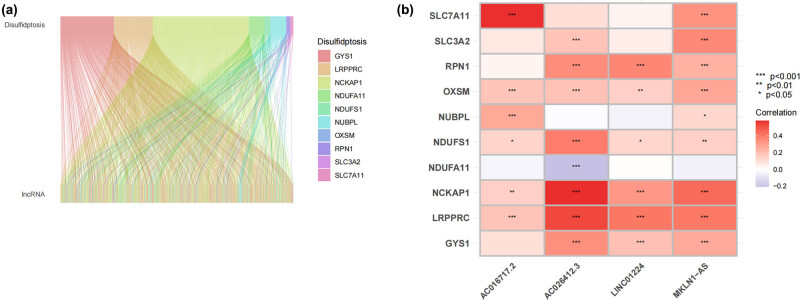
Connections between the disulfidptosis-related genes and dRlncRNAs. (a) Co-expression of ten genes involved in disulfidolysis and 717 dRlncRNAs, as shown by a Sankey diagram. (b) Ten genes involved in disulfidptosis were plotted against four dRlncRNAs in a heatmap indicating their relationship.

Within the TCGA_HCC dataset, we discerned a total of 16,201 lncRNAs. We further identified ten disulfidptosis-related genes. Through Pearson analysis, 717 dRlncRNAs and four prognostic-associated dRlncRNAs were uncovered. To illustrate the connections between the disulfidptosis-related genes and dRlncRNAs, we crafted Sankey diagrams and heatmaps (Figure 1a and b).

### 3.3 Construction and validation of dRlncRNAs risk model

**Figure 2:**
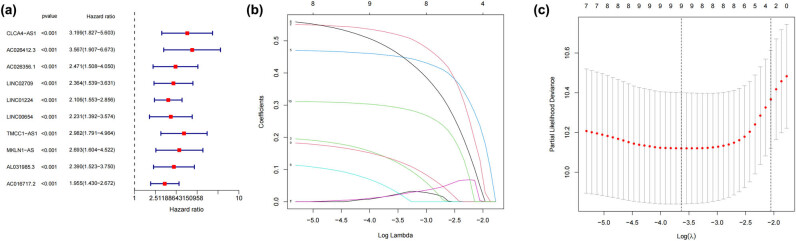
Identification of dRlncRNAs with prognostic value for HCC patients. (a) To identify prognostic dRlncRNAs, a univariate Cox regression analysis was used. (b) and (c) A prognostic prediction model was built using Lasso–Cox regression analysis.

**Figure 3:**
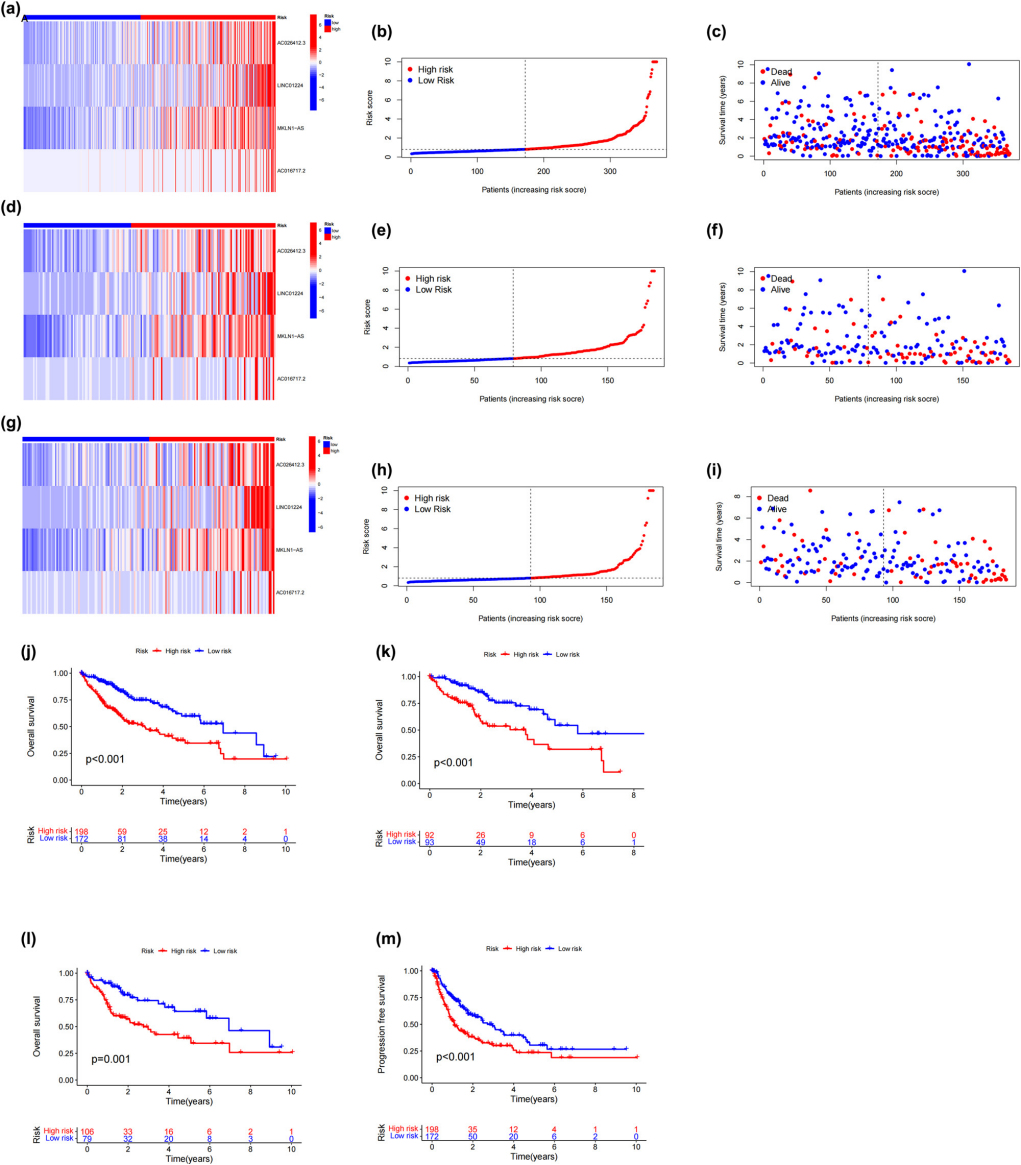
Model prediction of outcomes for different groups of patients. (a)–(c) The risk score distribution, survival status, and heatmap for high-risk and low-risk HCC patients within the entire cohort, (d)–(f) the training cohort, and (g)–(i) the testing cohort. (j)–(m) Both overall survival and progression-free survival were modeled using Kaplan–Meier curves.

Using univariate Cox regression analysis, researchers identified ten lncRNAs as potential survival predictors in patients with HCC (Figure 2a). As a next step, we used the LASSO regression technique (Figure 2b and c). Then, four dRlncRNAs were chosen as prognostic markers by a multivariate Cox regression analysis, and a risk model was developed. Figure 3a–i depicts the distribution of risk scores, which shows that there is an inverse association between survival time and increasing risk scores. It is also apparent from the data that the cohort of patients who were at a high risk had a much lower overall survival rate.

### 3.4 Risk model assessment

**Figure 4:**
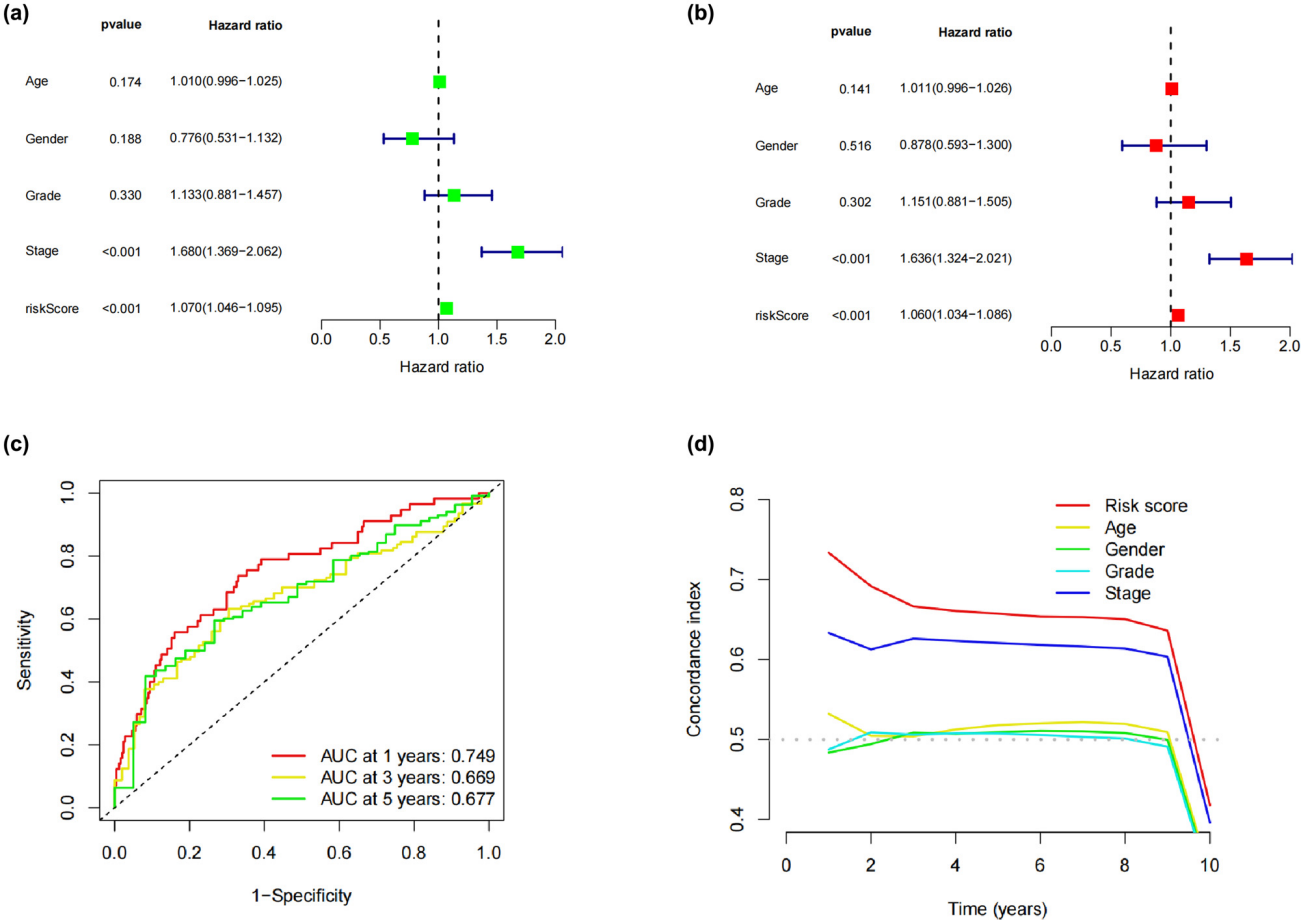
Independent prognostic analysis of the OS for HCC. (a) and (b) Analyses of risk of death by Cox proportional hazard models and their forest plots. (c) Predicting 1-year, 3-year, and 5-year OS in HCC patients using time-dependent ROC curves. (d) Compared to other clinical indicators, the risk model’s predictive accuracy is highlighted by a steeper c-index curve.

Both univariate and multivariate forms of the Cox regression analysis were utilized in the process of evaluating the predictive ability of the model. In univariate analysis, it was discovered that stage and risk score are both prognostic risk variables (Figure 4a and b), and in multivariate analysis, it was shown that risk score was a prognostic factor that could stand on its own. Figure 4c displays that the 1-year, 3-year, and 5-year areas under the ROC curve were 0.749, 0.669, and 0.677, respectively. The c-index (Figure 4d) showed that the predictive model’s accuracy was superior to that of other clinical parameters.

### 3.5 Column chart and PCA analysis

**Figure 5:**
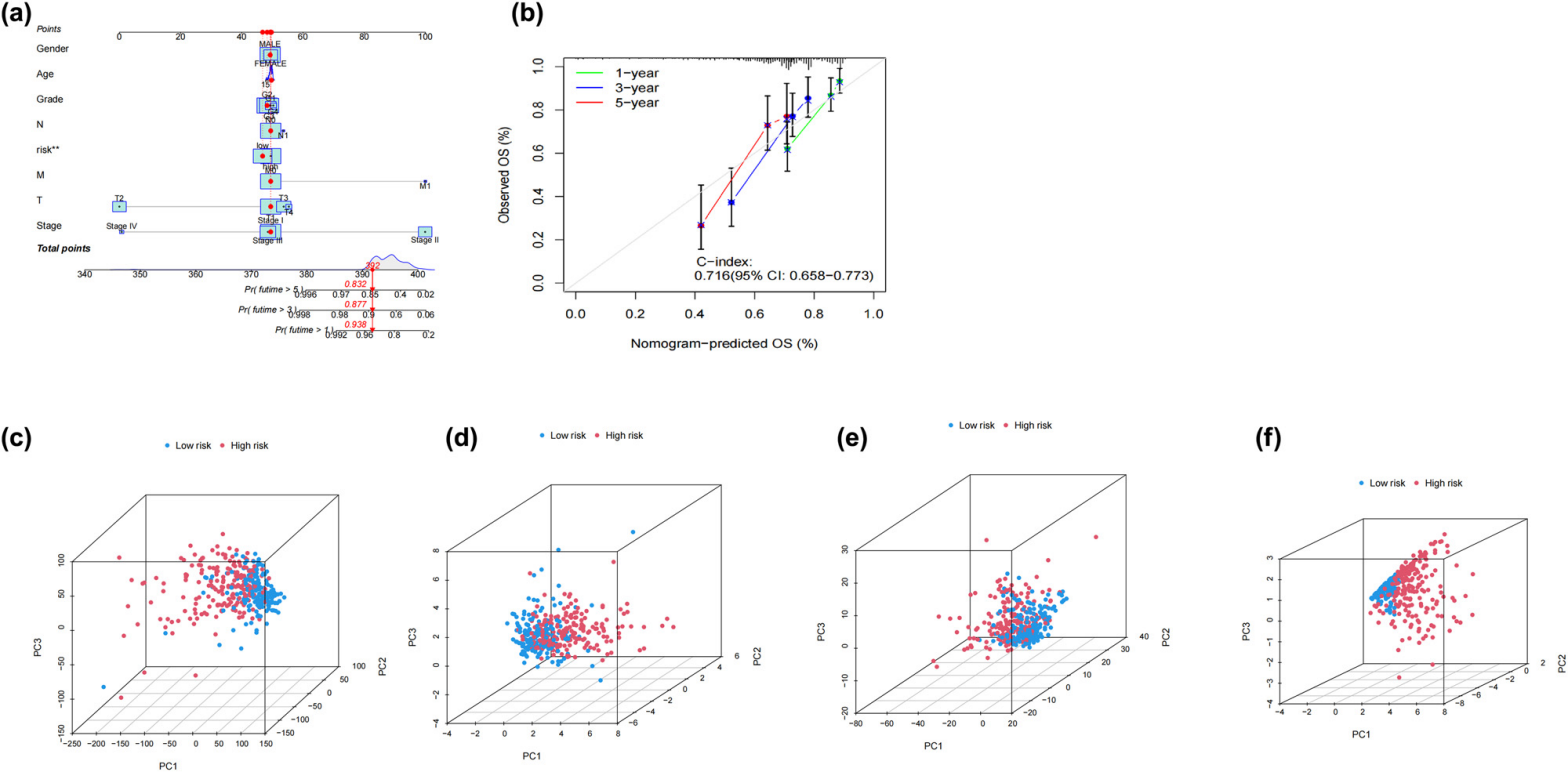
Validation of the nomogram model and PCA for predicting survival period. (a) The use of dRlncRNA as a predictive marker led to the development of a nomogram. (b) 1-, 3-, and 5-year survival prediction calibration plots. (c) PCA between the high-risk and low-risk groups based on all gene. (d) PCA between the high-risk and low-risk groups based on all disulfidptosis-related gene. (e) PCA between the high-risk and low-risk groups based on dRlncRNAs. (f) PCA between the high-risk and low-risk groups based on all risk lncRNAs.

As shown in Figure 5a, by combining several clinical indicators and risk scores into a nomogram model, we were able to improve our capacity to predict HCC patients’ 1-year, 3-year, and 5-year survival rates. Figure 5b shows that the predictions made by the column chart are in good agreement with the calibration chart. Subsequently, the 3D scatter plot (Figure 5c–f) displayed the unique clustering features of PCA in the low-risk and high-risk groups.

### 3.6 Functional enrichment analysis

**Figure 6:**
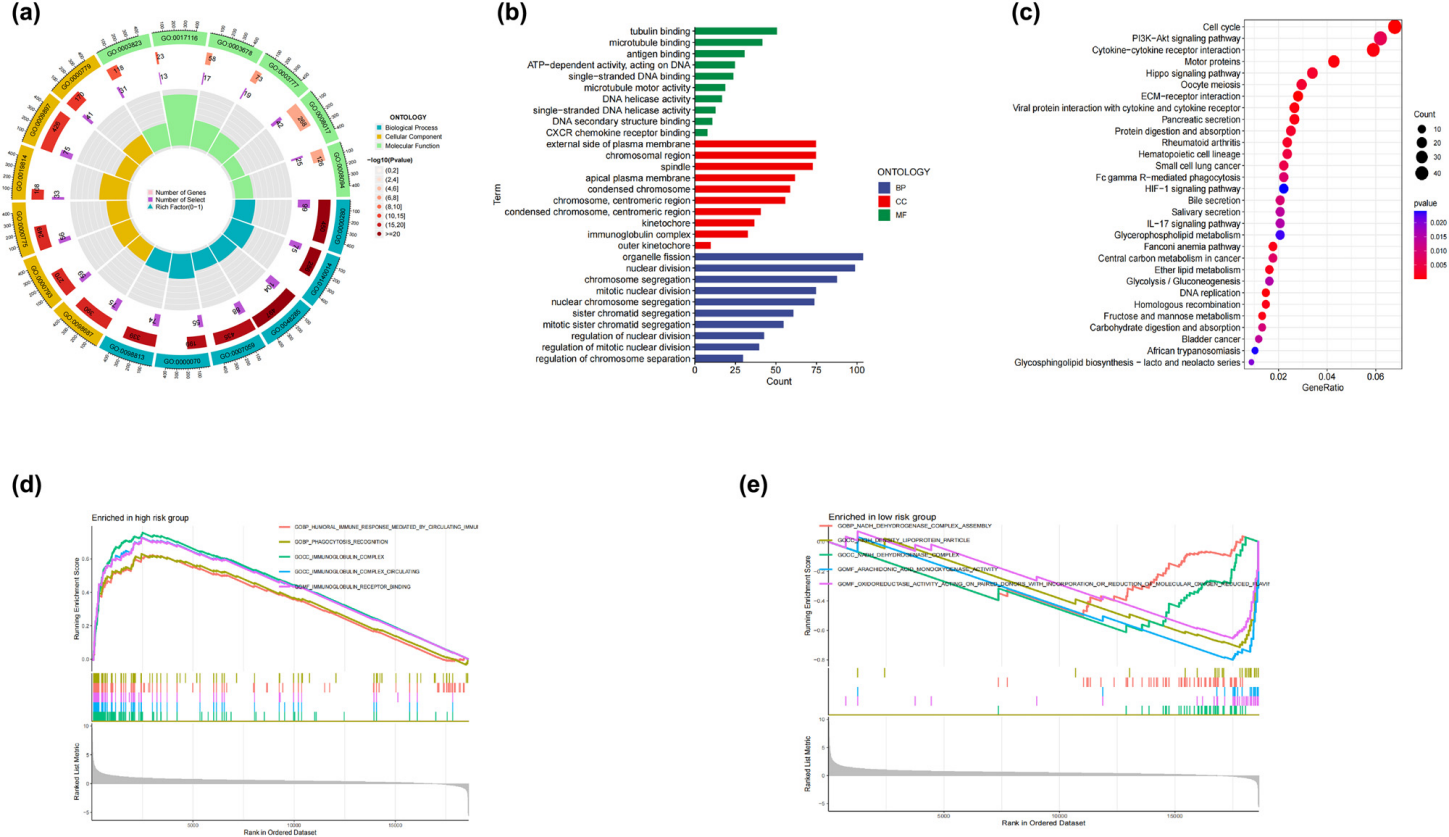
GO, KEGG, and GSEA enrichment analysis. (a) and (b) The outcomes of the GO enrichment analysis, providing insights into the functional annotations and biological processes associated with the identified genes. This analysis categorizes genes based on their involvement in cellular components, molecular functions, and biological processes, contributing to a comprehensive understanding of the biological implications. (c) The KEGG enrichment analysis results, offering a pathway-centric view of the identified genes. (d) High risk group GSEA enrichment analysis. (e) Low risk group GSEA enrichment analysis. GSEA provides a robust approach for evaluating the collective behavior of predefined gene sets, revealing potential associations with various biological functions and pathways.

In order to study the processes that are responsible for the major differences that exist between various risk groups, we carried out GO and KEGG enrichment analyses based on the DEGs that existed between the two groups. In the GO enrichment analysis, for the molecular function, the main enrichments were found in organelle fission; for the cellular component, the main enrichments were in the external side of plasma membrane and chromosomal region; and for the biological process, the main enrichments were in tubulin binding (Figure 6a and b). Additionally, the KEGG assays indicated that these DEGs were primarily enriched in the cell cycle pathway (Figure 6c).

We can see, through the use of GSEA, that groups with a high risk and groups with a low risk are enriched in quite different pathways. The high-risk group has a significantly higher abundance of immune-related pathways than the other groups. Among these are the humoral immune response that is mediated by circulating immunoglobulin, the recognition of phagocytosis by the immune system, the creation of immunoglobulin complexes, the circulation of immunoglobulin complexes, and the binding of immunoglobulin receptors (Figure 6d). Conversely, in the low-risk group, pathways involving processes like nicotinamide adenine dinucleotide (NADH) dehydrogenase complex assembly, high-density lipoprotein particle metabolism, NADH dehydrogenase complex activity, arachidonic acid monooxygenase activity, and oxidoreductase activity linked to oxygen incorporation or reduction exhibit notable enrichment. These findings suggest distinct molecular pathway patterns associated with different risk levels, shedding light on potential underlying mechanisms (Figure 6e).

### 3.7 Landscape analysis of immune infiltration

**Figure 7:**
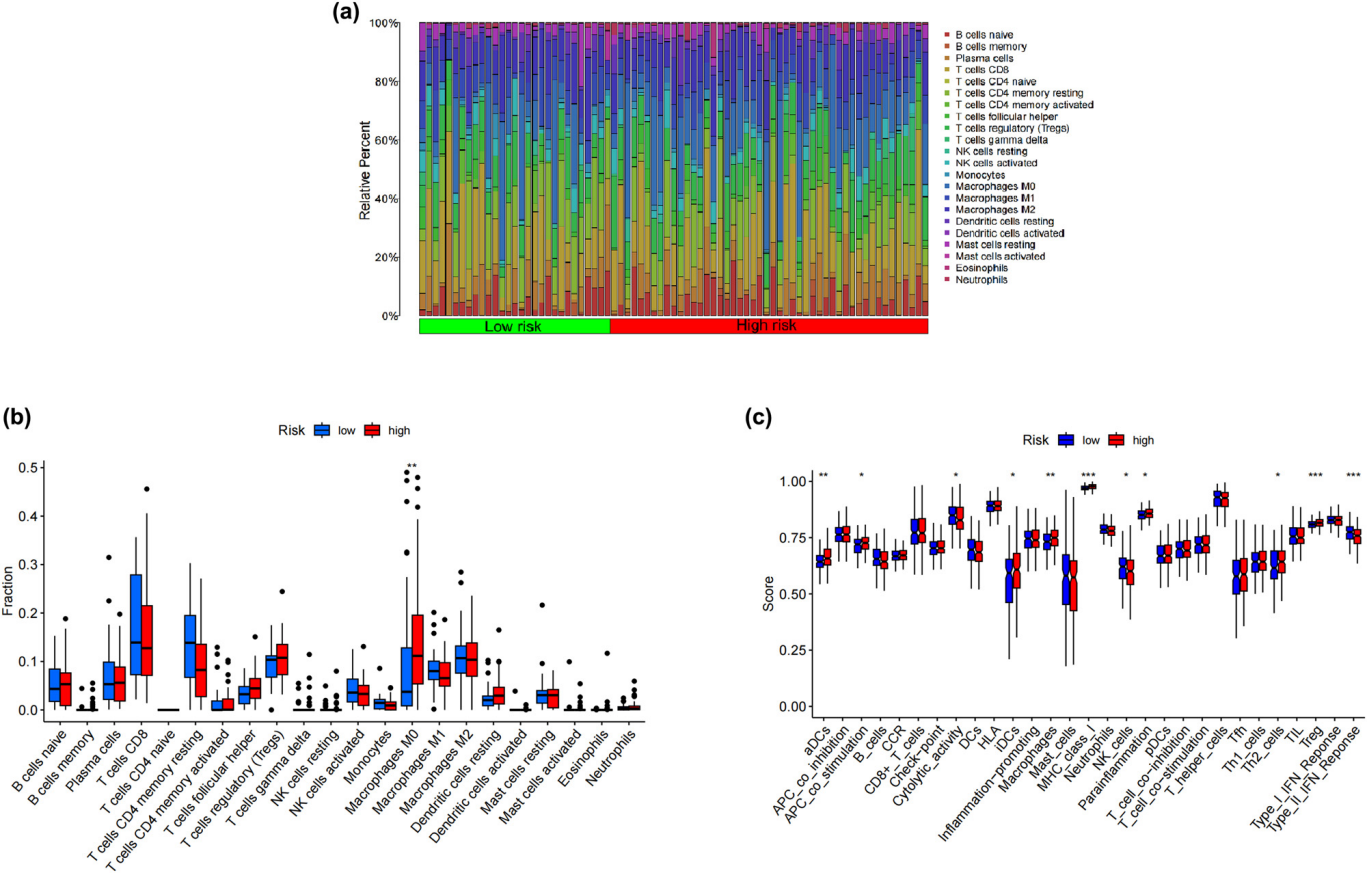
A detailed analysis of the immune infiltration landscape using advanced computational methods. (a) and (b) The CIBERSORT algorithm evaluates the differences between two groups of 22 immune cells. This analysis offers a granular understanding of the immune cell profile in each group, shedding light on potential variations in immune cell populations and their relative abundance. (c) The ssGSEA method compares the two groups’ immune cells and functions to determine their differences. By evaluating the enrichment of predefined gene sets associated with immune functions, ssGSEA provides insights into the overall immune landscape and highlights specific functional differences between the analyzed groups.

Using the CIBERSORT method, we characterized and compared 22 distinct immune cell types and their relative abundances. The results showed that the 22 different types of immune cells are distributed differently depending on the risk model (Figure 7a). Figure 7b’s boxplot reveals a significant rise in the percentage of M0 macrophages (*p* < 0.01). Additionally, we employed the ssGSEA algorithm to investigate immune cell infiltration and immune functionality across different risk groups. The results revealed notable variations in the proportions of immune cell populations. In the high-risk group, there were elevated proportions of aDCs (*p* < 0.01), APC co-stimulation (*p* < 0.05), iDCs (*p* < 0.05), macrophages (*p* < 0.05), MHC class I (*p* < 0.001), parainflammation (*p* < 0.05), Th2 cells (*p* < 0.05), and Treg (*p* < 0.001). Conversely, proportions of cytolytic activity (*p* < 0.05), NK cells (*p* < 0.05), and Type II IFN response (*p* < 0.001) were diminished. These findings shed light on the distinct immune cell profiles and functional shifts within varying risk populations (Figure 7c).

### 3.8 Tumor mutation burden and prognosis

**Figure 8:**
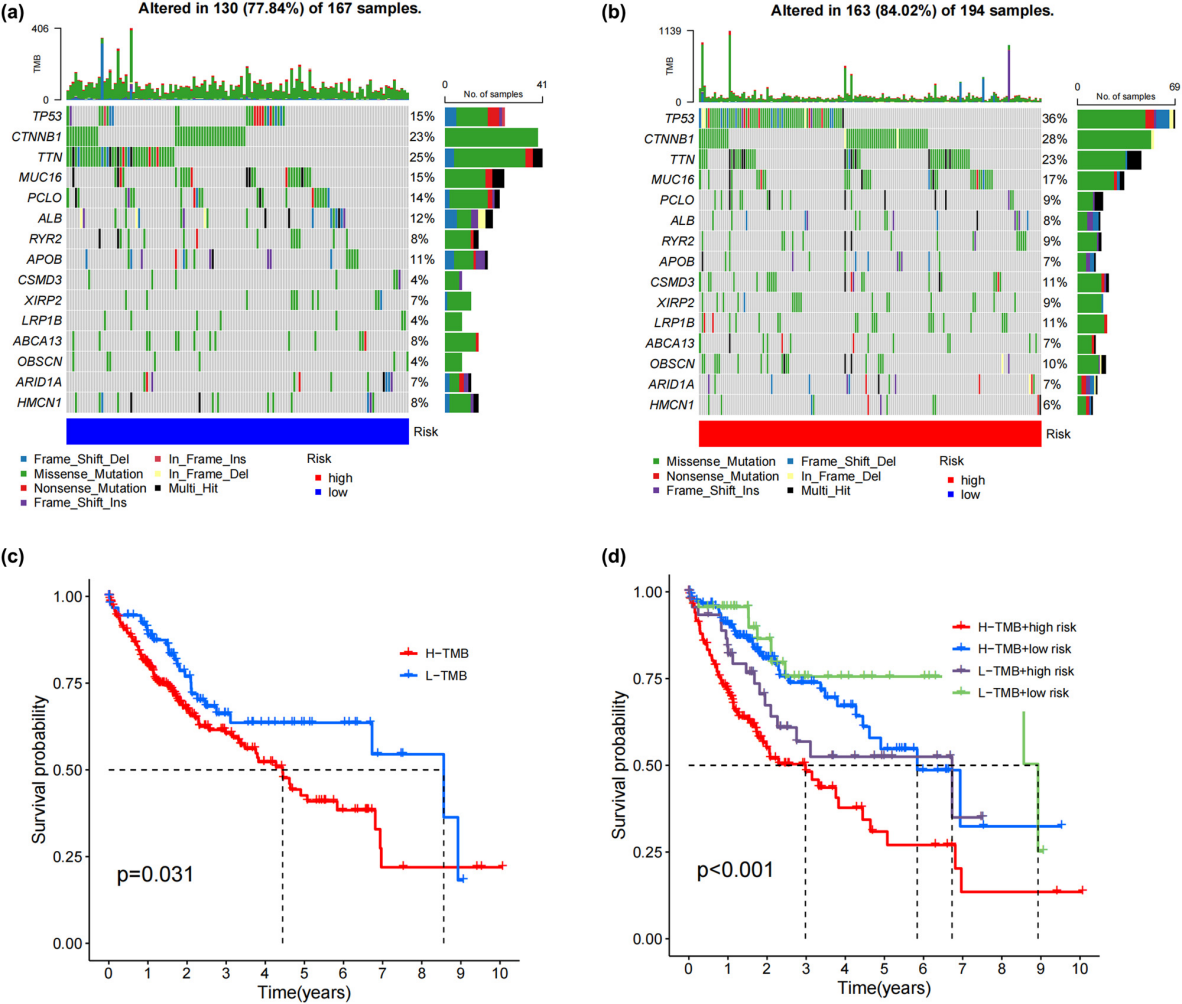
Tumor mutation burden and prognosis. (a) and (b) Distribution of mutations in patients in the low-risk and high-risk groups. (c) Survival probability between different TMB groups. (d) Survival probability between high and low TMB groups combined with high and low risk groups.

Comparisons were made between somatic mutations and a number of other types of potentially harmful effects. We observed mutations in 130 of 167 samples (77.84%) in the group with a low risk (Figure 8a), whereas in the group with a high risk, we found mutations in 163 of 194 samples (84.02%) (Figure 8b). We classified HCC patients into low TMB and high TMB groups based on the median TMB scores. The low TMB group had a much better survival rate than the high TMB group (Figure 8c). Our ultimate strategy involved the integration of risk scores and TMB scores to enhance the precision of prognosis estimation for HCC patients and ascertain the superior predictive value between the two scores. The Kaplan–Meier analysis results further elucidated these findings (shown in Figure 8d). The overall survival rates are at their best for patients who have low TMB and low risk scores, while they are at their worst for individuals who have high TMB and high risk scores.

### 3.9 Drug sensitivity analysis

**Figure 9:**
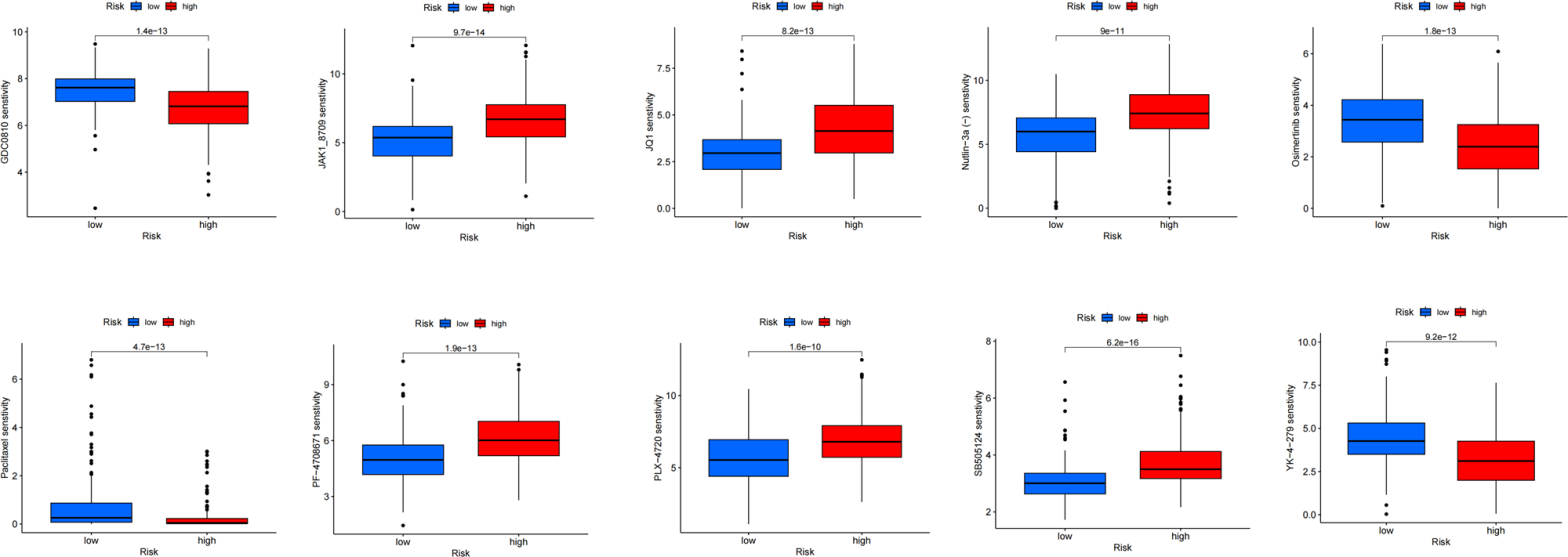
Drug sensitivity analysis. IC_50_ correlated with high and low risk patients in HCC.

We further explore the possibility of dRlncRNA as a predictive marker for customized therapy in HCC by comparing drug risk ratings to IC_50_ values in the treatment of HCC. Figure 9 displays a statistically significant (*p* < 0.01) difference in sensitivity to ten different classes of anti-cancer medications between the two groups. Potentially, these medicines will be used in the future to treat HCC. Low-risk HCC patients may be a better fit for the medicines JAK1_8709, JQ1, Nutlin-3a (−), PF-4708671, PLX-4720, and SB505124, as their IC_50_ values are lower. Patients with HCC who are at a higher risk respond better to treatment with GDC0810, Osimertinib, Paclitaxel, and YK-4-279, as shown by a lower value in the high-risk group (Figure 9).

## 4 Discussion

As liver cancer treatment has progressed, a wide range of therapeutic strategies, such as liver transplantation, surgical resection, systemic treatments, and targeted therapies, have been consistently improved and innovated [12]. Presently, surgical intervention stands as the potential curative approach for HCC. However, only 15% of HCC patients are eligible for surgical treatment, given that the majority of cases are diagnosed at an advanced stage [13]. Immunotherapy targeting disulfidaptosis stands out as a novel and promising avenue in the realm of cancer treatment, offering broader application possibilities. Unlike apoptosis and ferroptosis, this unique form of cell demise known as disulfidaptosis remains unmitigated by conventional cell death inhibitors and is distinct from ATP depletion-induced mechanisms. Instead, it is amplified by thiol oxidizing agents like dithiothreitol [14]. Metabolic interventions involving the inhibition of glucose transporter proteins can trigger the accumulation of disulfide bonds, effectively impeding the growth of various cancers, including renal cell carcinoma [15].

The lncRNA genes are independent transcription units that can be transcribed into non-coding RNA with a length of 200 nucleotides [16,17]. LncRNA plays a pivotal role in tumor development and immune response. Within the TME, lncRNA exhibits unique cellular functions [17]. In this study, four lncRNAs associated with disulfidptosis prognosis were constructed into a predictive model and used to validate the prognosis of HCC patients. There have been reports in the literature regarding AC026412.3, LINC01224, and MKLN1-AS, but there is no report about AC016717.2 [18–20].

To learn more about the DRlncRNAs prognostic model’s biological roles and signaling pathways, we performed an enrichment analysis. The GO and KEGG results suggest that DRlncRNAs are associated with various immune and metabolic pathways, primarily involving cell division, cell growth, cell signaling, gene expression, and regulation of the cell cytoskeleton. GSEA analysis suggests a potential involvement of DRlncRNAs in the function and regulation of pathways. The high-risk group demonstrated pathway enrichment associated with immune response, potentially implicating immune cell activity and inflammatory reactions. Conversely, the low-risk group exhibited pathway enrichment linked to energy metabolism, lipid metabolism, and inflammatory regulation. In summary, these findings reveal the significance of DRlncRNAs in regulating immune and metabolic processes. Results from the high-risk group suggest potential involvement of DRlncRNAs in immune responses, possibly associated with biological processes related to inflammation. Conversely, outcomes from the low-risk group indicate that DRlncRNAs may play a crucial role in regulating energy metabolism and lipid metabolism. These results provide valuable clues for further exploring the biological functions of DRlncRNAs and identifying potential therapeutic targets.

The current landscape of HCC treatment is confronted with significant challenges primarily stemming from the intricate and diverse nature of the immune microenvironment [21]. The complexity arises from the multifaceted interactions among various immune cell populations, stromal components, and tumor cells within the HCC microenvironment [22]. This heterogeneity poses a substantial obstacle in devising effective therapeutic strategies, as the dynamic interplay of immune responses, inflammatory processes, and immunosuppressive mechanisms contributes to the intricate network of tumor–host interactions [23–25]. Addressing these challenges requires a comprehensive understanding of the immune landscape in HCC, considering factors such as tumor-infiltrating lymphocytes, immune checkpoint regulation, and the influence of the TME on treatment responses. Therefore, unraveling the complexities and heterogeneities of the immune microenvironment in HCC is crucial for developing tailored and effective therapeutic interventions. In the high-risk group of HCC, there is elevated expression of macrophages M0. This finding aligns with the study by Yang et al.: in intrahepatic cholangiocarcinoma, M0 macrophages were significantly increased compared to the pericancerous tissue [26]. In this study, we utilized the ssGSEA algorithm to investigate immune cell infiltration and immune functionality across different risk groups, uncovering significant variations in the proportions of immune cell populations. Elevated proportions of aDCs, APC co-stimulation, iDCs, macrophages, MHC class I, parainflammation, Th2 cells, and Treg were observed in the high-risk group, juxtaposed with a reduction in cytolytic activity, NK cells, and Type II IFN response. These findings shed light on the unique immune cell profiles and functional shifts within varying risk populations, potentially relating to disease progression, prognosis, and therapeutic responses. They lay essential groundwork for the future development of more targeted immunotherapeutic strategies. Further research is warranted to delve into the underlying mechanisms of these changes and validate the potential clinical utility of these insights.

Our research indicated that TP53 mutations are more common in HCC. In HCC, changes in TP53 are associated with serum alpha-fetoprotein levels, tumor staging, vascular invasion, tumor differentiation, and Child-Pugh classification [27–30]. The risk model built using dRlncRNAs has a substantial positive connection with TMB, and a greater TMB is linked to worse survival outcomes. This provides more evidence that the risk score was a useful prediction tool for HCC patients undergoing immunotherapy. We also assessed the sensitivity of anticancer medicines in patients with HCC across multiple risk categories, yielding novel recommendations for the treatment of a wide range of HCC cases.

Our research has unearthed a nuanced relationship between the sensitivity of specific drugs and the risk profiles of HCC patients. The observed lower IC_50_ values for JAK1_8709, JQ1, Nutlin-3a (−), PF-4708671, PLX-4720, and SB505124 in the low-risk group, and GDC0810, Osimertinib, Paclitaxel, and YK-4-279 in the high-risk group, illuminate a path toward more targeted therapeutic interventions. These findings indicate that HCC treatment could benefit from a stratified approach, wherein patients are classified into risk groups, and medications are selected accordingly. Such an approach does not merely represent a refinement of treatment selection; it potentially heralds a paradigm shift in patient management, emphasizing the importance of individualized care based on a comprehensive understanding of the disease risk. It also opens up avenues for further research, including the validation of these findings through clinical trials, and exploring the underlying biological mechanisms that might explain the observed differences in drug sensitivity among different risk groups. This could be a pivotal step in improving both the efficacy and efficiency of HCC treatment protocols.

This study has certain limitations. First, the study was primarily conducted in public databases. Further validation in additional clinical cases is required. Second, the potential function of lncRNAs was not confirmed *in vitro* and *in vivo*.

## 5 Conclusions

In summary, this study is the first in HCC to focus on DRlncRNA. By constructing a prognostic model through the regulation of lncRNA in the disulfidptosis process, it brings prospects for the diagnosis and treatment of HCC patients. Overall, this study not only introduces DRlncRNAs as potential prognostic markers for HCC but also lays the foundation for further investigations into the regulatory networks governing disulfidptosis. The findings hold promise for advancing both diagnostic and therapeutic strategies in HCC, making a substantial contribution to the ongoing efforts to combat this challenging and prevalent form of HCC.
